# Crystal structure of 9,20-dimethyl-1,8,12,19-tetra­aza­tetra­cyclo­[17.3.1.0^2,7^.0^13,18^]tricosane dihydrate from synchrotron X-ray data

**DOI:** 10.1107/S2056989017002444

**Published:** 2017-02-17

**Authors:** Dohyun Moon, Jong-Ha Choi

**Affiliations:** a Pohang Accelerator Laboratory, POSTECH, Pohang 37673, Republic of Korea; b Department of Chemistry, Andong National University, Andong 36729, Republic of Korea

**Keywords:** crystal structure, macropolycycle, 1,3-di­aza­cyclo­hexane ring, hydrogen bonds, synchrotron radiation

## Abstract

The structure of the title compound, C_21_H_40_N_4_·2H_2_O, has been determined from synchrotron X-ray radiation data. The asymmetric unit comprises one 12-membered macropolycycle and two lattice water mol­ecules. The macropolycycle contains two cyclo­hexane rings and one 1,3-di­aza­cyclo­hexane ring, all in chair conformations. The C—N and C—C bond lengths are in the ranges 1.4526 (16)–1.4786 (17) and 1.517 (2)–1.5414 (17) Å, respectively. One intra­molecular N—H⋯N hydrogen bond helps to stabilize the mol­ecular conformation while medium-strength inter­molecular N—H⋯O, O—H⋯N and O—H⋯O hydrogen bonds involving the lattice water mol­ecules connect the components into a three-dimensional network.

## Abstract 2

The macrocyclic title compound crystallizes as a dihydrate with a 12-membered inner ring system. Hydrogen bonds involving the lattice water mol­ecules link the components into a three-dimensional network system.

## Chemical context   

Macrocyclic ligands and their complexes are involved in diverse application fields such as catalysis, enzyme mimics, chemical sensors, purification of waste water, selective metal-ion recovery and anti­tumor agents and therapy (Meyer *et al.*, 1998 ▸). The family of macrocyclic amines with fourteen-membered inner rings has received attention due to their anti-HIV activity (Liang & Sadler, 2004 ▸; Ronconi & Sadler, 2007 ▸; Ross *et al.*, 2012 ▸). There has also been considerable inter­est in *C-* or *N*-functionalized macrocyclic compounds and their metal complexes because the structural and chemical properties are often quite different from those of the corres­ponding non-functionalized compounds (Barefield, 2010 ▸; Choi *et al.*, 2010 ▸). Structural modifications of the macrocycles based on methyl­ene bridging of adjacent nitro­gen atoms have been achieved using various methods (Royal *et al.*, 1998 ▸; Tripier *et al.*, 2001 ▸; Hubin, 2003 ▸; Kang *et al.*, 2008 ▸).
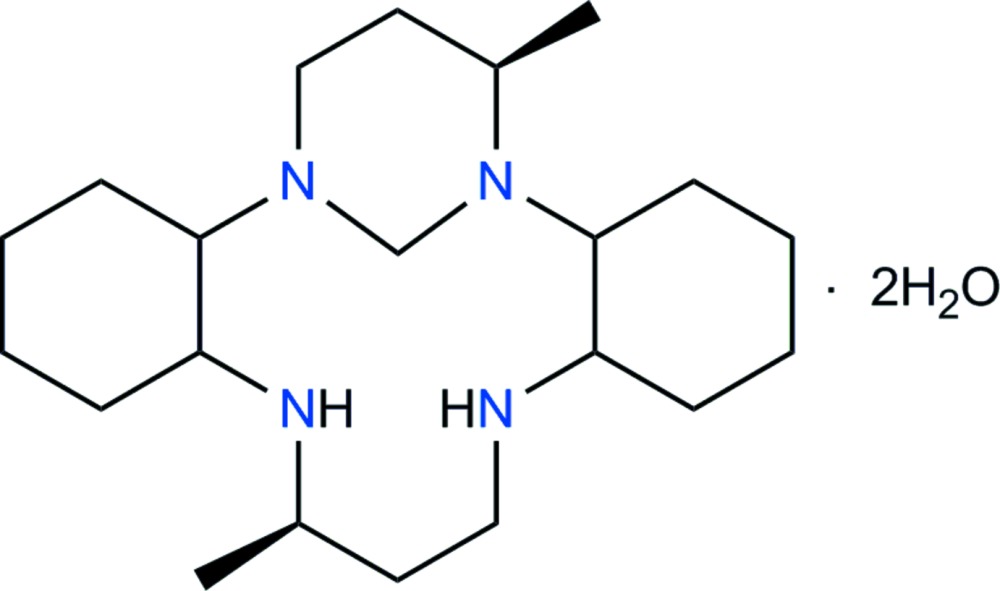


The synthesis of the 9,20-dimethyl-1,8,12,19-tetra­aza­tetra­cyclo­[17.3.1.0^2,7^.0^13,18^]tricosane (*L* ^2^) monohydrated com­pound, *L* ^2^·H_2_O has been described previously (Kang *et al.*, 2008 ▸), but we could not obtain a suitable single crystal of this compound for structure determination using X-ray diffraction. Formaldehyde has been utilized for the synthesis of such polyaza macrocyclic and macropolycyclic compounds containing five- or six-membered rings. We recently described the preparation, spectroscopic properties and the mol­ecular and crystal structure of 3,14-dimethyl-2,6,13,17-tetra­aza­penta­cyclo­(16.4.1^2,17^.1^6,13^.0.0^7,12^)tetra­cosane containing two 1,3-di­aza­cyclo­pentane rings, which was the major product from the reaction of 3,14-dimethyl-2,6,13,17-tetra­aza­tri­cyclo­(16.4.0^1,18^.0^7,12^)docosane (*L* ^1^) with two equivalents of formaldehyde (Moon *et al.*, 2016 ▸).

In the present work, we attempted the reaction of *L* ^1^ with one equivalent of formaldehyde and synthesized the title compound, C_21_H_40_N_4_·2H_2_O, (I). Inter­estingly, the title compound, containing a six-membered 1,3-di­aza­cyclo­hexane ring, was the main product of the synthesis, while the compound containing a five-membered 1,3-di­aza­cyclo­pentane ring did not crystallize. In order to determine the mol­ecular and crystal structure of the title compound, single-crystal X-ray structural determination was performed by using synchrotron data.

## Structural commentary   

**Figure 1:**
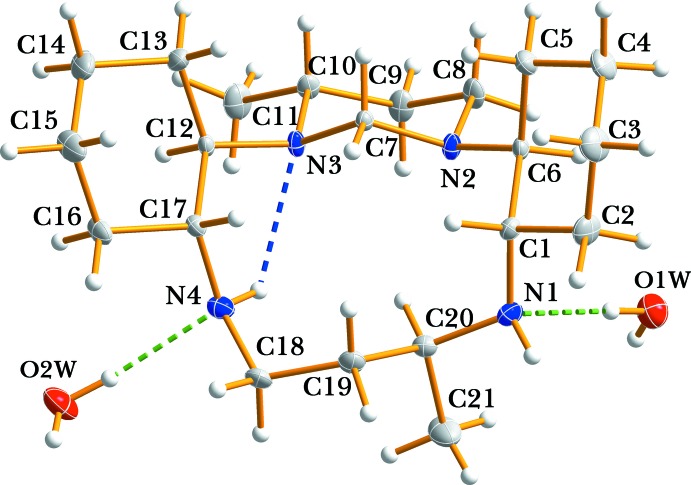
The asymmetric unit of (I), showing the atom-numbering scheme. Non-H atoms are shown as displacement ellipsoids at the 50% probability level. Hydrogen-bonding inter­actions are indicated by dashed lines.

Fig. 1 ▸ shows an ellipsoid plot of the mol­ecular components of compound (I). The asymmetric unit comprises a macrocyclic C_21_H_40_N_4_ mol­ecule and two lattice water mol­ecules. The two methyl substituents of the C10 and C20 atoms are on the same side with respect to the macrocyclic plane of the four N atoms (Fig. 1 ▸). The cyclo­hexane rings, together with the 1,3-di­aza­cyclo­hexane ring and the 1,3- di­amino-1-methyl­propane moiety, are fused to the 12-membered macrocycle. All six-membered rings exist in a slightly distorted chair conformation. The N1—C1—C6—N2 and N3—C12—C17—N4 torsion angle displays a *gauche* conformation. The bond lengths are in the ranges 1.4526 (16)–1.4786 (17) Å and 1.517 (2)–1.5414 (17) Å for the C—N and C—C bonds, respectively. The N1—C20 distance is the longest C—N distance, presumably as a consequence of the methyl group on the C20 atom and the N⋯H—O hydrogen bond involving N1. The bond angles within the six-membered 1,3-di­aza­cyclo­hexane ring, N2—C7—N3, C7—N2—C8, and C7—N3—C10, are 109.89 (10), 109.60 (10), and 108.08 (9)°, respectively. All other C—N, C—C, and C—H bond lengths and corresponding angles are in the normal range for such compounds (Royal *et al.*, 1998 ▸; Tripier *et al.*, 2001 ▸). The intra­molecular hydrogen bond between the amine group N4—H1*N*4 and the tertiary N3 atom lends some rigidity to the 12-membered macropolycycle *L* ^2^ ring (Fig. 1 ▸).

## Supra­molecular features   

**Figure 2:**
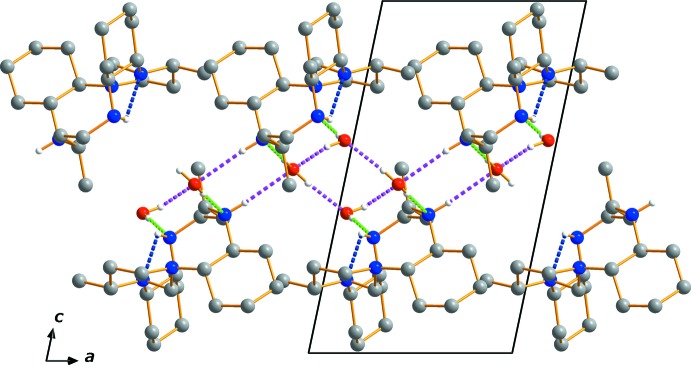
Crystal packing diagram of (I), viewed perpendicular to the *ac* plane. H atoms not involved in hydrogen bonds have been omitted. The dashed lines represent N—H⋯N (blue), O—H⋯O (pink) and O—H⋯N (green) hydrogen bonds, respectively.

In the crystal, the macropolycycle and the two water mol­ecules are held together by N—H⋯O, and O—H⋯O hydrogen bonds (Table 1 ▸); O—H⋯O hydrogen bonds between the water mol­ecules are also observed. All inter­molecular hydrogen-bonding inter­actions are of medium strength and lead to the formation of a three-dimensional network between the components. The packing along the *b* axis is shown in Fig. 2 ▸.

## Database survey   

A search of the Cambridge Structural Database (Version 5.37, May 2016 with three updates; Groom *et al.* 2016 ▸) gave just one hit for a 9,20-dimethyl-1,8,12,19-tetra­aza­tetra­cyclo[17.3.1.0^2,7^.0^13,18^]tricosane (*L* ^2^) unit, *viz*. the crystal structure of [Cr(*L* ^2^)(H_2_O)](ClO_4_)_2_·3H_2_O (Kang *et al.*, 2008 ▸). However, no structure of any other compound with *L* ^2^ has been deposited.

## Synthesis and crystallization   

Commercially available (Sigma–Aldrich) 1,2-cyclo­hexa­nedi­amine was used as provided. All other chemicals were reagent grade and used without further purification. The starting material, 3,14-dimethyl-2,6,13,17-tetra­aza­tri­cyclo(16.4.0^1,18^.0^7,12^)docosane (*L* ^1^) was synthesized according to a literature protocol (Kang & Jeong, 2003 ▸). To a solution of *L* ^1^ (0.5 g, 1.5 mmol) in H_2_O (40 mL) was rapidly added 37% formaldehyde (0.11 mL, 1.5 mmol) at room temperature. The reaction mixture was refluxed for 3 h. After cooling, the resultant white solid was filtered, washed with water, and dried. The crude product of *L* ^2^·2H_2_O, (I), was recrystallized from a hot THF/H_2_O (1:2 *v*/*v*) solution to give colourless crystals suitable for X-ray analysis.

## Refinement   

Crystal data, data collection, and structure refinement details are summarized in Table 2 ▸. All C-bound H atoms in the complex were placed in geometrically idealized positions and constrained to ride on their parent atoms, with C—H distances of 0.98–1.00 Å with *U*_iso_(H) values of 1.5 and 1.2 *U*_eq_ of the parent atoms, respectively. N- and O-bound H atoms were assigned based on a difference Fourier map, and were refined with distance restraints of 0.91 (4) and 0.88 (2) Å (using DFIX and DANG commands), respectively, and with *U*_iso_(H) values of 1.2*U*_eq_ of the parent atoms.

## Supplementary Material

Crystal structure: contains datablock(s) I. DOI: 10.1107/S2056989017002444/wm5364sup1.cif

Structure factors: contains datablock(s) I. DOI: 10.1107/S2056989017002444/wm5364Isup2.hkl

Supporting information file. DOI: 10.1107/S2056989017002444/wm5364Isup3.cml

CCDC reference: 1532347

Additional supporting information:  crystallographic information; 3D view; checkCIF report

## Figures and Tables

**Table 1:** Hydrogen-bond geometry (Å, °)

*D*—H⋯*A*	*D*—H	H⋯*A*	*D*⋯*A*	*D*—H⋯*A*
N1—H1*N*1⋯O1*W* ^i^	0.914 (15)	2.395 (15)	3.2763 (19)	162.0 (13)
N4—H1*N*4⋯N3	0.882 (16)	2.287 (15)	2.8304 (16)	119.8 (12)
O1*W*—H1*O*1⋯N1	0.85 (1)	2.06 (1)	2.9077 (18)	178 (2)
O1*W*—H2*O*1⋯O2*W* ^ii^	0.83 (1)	1.99 (1)	2.816 (2)	175 (2)
O2*W*—H1*O*2⋯N4	0.84 (1)	2.10 (1)	2.9190 (18)	168 (2)
O2*W*—H2*O*2⋯O1*W* ^iii^	0.83 (1)	1.98 (1)	2.7975 (17)	166 (2)

Symmetry codes: (i) 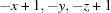; (ii) 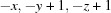; (iii) 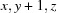.

**Table 2:** Experimental details

Crystal data	
Chemical formula	C_21_H_40_N_4_·2H_2_O
*M*_r_	384.60
Crystal system, space group	Triclinic, *P* 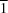
Temperature (K)	173
*a*, *b*, *c* (Å)	8.3870 (17), 10.275 (2), 14.115 (3)
α, β, γ (°)	87.20 (3), 77.83 (3), 72.31 (3)
*V* (Å^3^)	1132.8 (5)
*Z*	2
Radiation type	Synchrotron, λ = 0.610 Å
μ (mm^−1^)	0.06
Crystal size (mm)	0.08 × 0.07 × 0.05

Data collection	
Diffractometer	ADSC Q210 CCD area detector
Absorption correction	Empirical (using intensity measurements) (*HKL3000sm *SCALEPACK**; Otwinowski & Minor, 1997 ▸)
*T*_min_, *T*_max_	0.878, 1.000
No. of measured, independent and observed [*I* > 2σ(*I*)] reflections	11640, 5950, 3853
*R*_int_	0.045
(sin θ/λ)_max_ (Å^−1^)	0.719

Refinement	
*R*[*F* ^2^ > 2σ(*F* ^2^)], *wR*(*F* ^2^), *S*	0.047, 0.123, 0.92
No. of reflections	5950
No. of parameters	265
No. of restraints	6
H-atom treatment	H atoms treated by a mixture of independent and constrained refinement
Δρ_max_, Δρ_min_ (e Å^−3^)	0.37, −0.27

Computer programs: *PAL BL2D-SMDC* (Shin *et al.*, 2016 ▸), *HKL3000sm* (Otwinowski & Minor, 1997 ▸), *SHELXT2014* (Sheldrick, 2015*a* ▸), *SHELXL2014* (Sheldrick, 2015*b* ▸), *DIAMOND 4* (Putz & Brandenburg, 2014 ▸) and *publCIF* (Westrip, 2010 ▸).

## supplementary crystallographic information

### Crystal data

**Table:**

C_21_H_40_N_4_·2H_2_O	*Z* = 2
*M**_r_* = 384.60	*F*(000) = 428
Triclinic, *P*1	*D*_x_ = 1.128 Mg m^−^^3^
*a* = 8.3870 (17) Å	Synchrotron radiation, λ = 0.610 Å
*b* = 10.275 (2) Å	Cell parameters from 63772 reflections
*c* = 14.115 (3) Å	θ = 0.4–33.6°
α = 87.20 (3)°	µ = 0.06 mm^−^^1^
β = 77.83 (3)°	*T* = 173 K
γ = 72.31 (3)°	Block, colorless
*V* = 1132.8 (5) Å^3^	0.08 × 0.07 × 0.05 mm

### Data collection

**Table:**

ADSC Q210 CCD area detector diffractometer	3853 reflections with *I* > 2σ(*I*)
Radiation source: PLSII 2D bending magnet	*R*_int_ = 0.045
ω scan	θ_max_ = 26.0°, θ_min_ = 1.3°
Absorption correction: empirical (using intensity measurements) (*HKL3000sm SCALEPACK*; Otwinowski & Minor, 1997)	*h* = −11→11
*T*_min_ = 0.878, *T*_max_ = 1.000	*k* = −14→14
11640 measured reflections	*l* = −18→18
5950 independent reflections	

### Refinement

**Table:**

Refinement on *F*^2^	Hydrogen site location: mixed
Least-squares matrix: full	H atoms treated by a mixture of independent and constrained refinement
*R*[*F*^2^ > 2σ(*F*^2^)] = 0.047	*w* = 1/[σ^2^(*F*_o_^2^) + (0.0685*P*)^2^] where *P* = (*F*_o_^2^ + 2*F*_c_^2^)/3
*wR*(*F*^2^) = 0.123	(Δ/σ)_max_ < 0.001
*S* = 0.92	Δρ_max_ = 0.37 e Å^−^^3^
5950 reflections	Δρ_min_ = −0.27 e Å^−^^3^
265 parameters	Extinction correction: SHELXL2014 (Sheldrick, 2015*b*), Fc^*^=kFc[1+0.001xFc^2^λ^3^/sin(2θ)]^-1/4^
6 restraints	Extinction coefficient: 0.042 (4)

### Special details

**Table:**

Geometry. All esds (except the esd in the dihedral angle between two l.s. planes) are estimated using the full covariance matrix. The cell esds are taken into account individually in the estimation of esds in distances, angles and torsion angles; correlations between esds in cell parameters are only used when they are defined by crystal symmetry. An approximate (isotropic) treatment of cell esds is used for estimating esds involving l.s. planes.

### Fractional atomic coordinates and isotropic or equivalent isotropic displacement parameters (Å^2^)

**Table:**

	*x*	*y*	*z*	*U*_iso_*/*U*_eq_	
N1	0.44588 (14)	0.16840 (11)	0.39264 (8)	0.0138 (2)	
H1N1	0.532 (2)	0.1462 (15)	0.4261 (11)	0.017*	
N2	0.24153 (13)	0.20083 (10)	0.24205 (8)	0.0101 (2)	
N3	0.11157 (13)	0.43397 (10)	0.20720 (8)	0.0094 (2)	
N4	0.19952 (14)	0.60232 (11)	0.32752 (8)	0.0129 (2)	
H1N4	0.138 (2)	0.5454 (16)	0.3368 (11)	0.015*	
C1	0.52453 (16)	0.12714 (13)	0.29073 (9)	0.0118 (3)	
H1	0.5594	0.2047	0.2563	0.014*	
C2	0.68449 (18)	0.00477 (14)	0.28851 (11)	0.0200 (3)	
H2A	0.7651	0.0318	0.3197	0.024*	
H2B	0.6516	−0.0698	0.3268	0.024*	
C3	0.77460 (18)	−0.04848 (15)	0.18675 (11)	0.0241 (3)	
H3A	0.8724	−0.1306	0.1902	0.029*	
H3B	0.8199	0.0219	0.1503	0.029*	
C4	0.65341 (19)	−0.08452 (14)	0.13344 (11)	0.0222 (3)	
H4C	0.6182	−0.1622	0.1654	0.027*	
H4D	0.7124	−0.1123	0.0658	0.027*	
C5	0.49601 (18)	0.03878 (13)	0.13402 (10)	0.0165 (3)	
H5A	0.4168	0.0139	0.1004	0.020*	
H5B	0.5314	0.1139	0.0980	0.020*	
C6	0.40204 (16)	0.08885 (12)	0.23757 (9)	0.0109 (2)	
H6	0.3714	0.0099	0.2724	0.013*	
C7	0.26051 (15)	0.31402 (12)	0.17952 (9)	0.0097 (2)	
H7A	0.2718	0.2882	0.1112	0.012*	
H7B	0.3654	0.3357	0.1852	0.012*	
C8	0.09840 (17)	0.15840 (13)	0.22515 (10)	0.0159 (3)	
H8A	0.0817	0.0839	0.2696	0.019*	
H8B	0.1225	0.1242	0.1577	0.019*	
C9	−0.06214 (17)	0.28106 (14)	0.24294 (11)	0.0176 (3)	
H9A	−0.0941	0.3061	0.3127	0.021*	
H9B	−0.1574	0.2555	0.2258	0.021*	
C10	−0.03765 (16)	0.40486 (13)	0.18391 (10)	0.0132 (3)	
H10	−0.0115	0.3802	0.1135	0.016*	
C11	−0.19966 (17)	0.52625 (15)	0.20482 (11)	0.0222 (3)	
H11A	−0.2158	0.5617	0.2705	0.033*	
H11B	−0.2983	0.4969	0.1996	0.033*	
H11C	−0.1889	0.5981	0.1578	0.033*	
C12	0.14475 (16)	0.56142 (12)	0.16863 (9)	0.0106 (2)	
H12	0.0332	0.6355	0.1839	0.013*	
C13	0.20962 (19)	0.56205 (14)	0.05870 (10)	0.0177 (3)	
H13A	0.3206	0.4899	0.0406	0.021*	
H13B	0.1274	0.5408	0.0255	0.021*	
C14	0.2320 (2)	0.70002 (15)	0.02457 (11)	0.0253 (3)	
H14A	0.2810	0.6949	−0.0459	0.030*	
H14B	0.1191	0.7708	0.0359	0.030*	
C15	0.3498 (2)	0.73944 (16)	0.07885 (11)	0.0266 (4)	
H15A	0.4663	0.6744	0.0613	0.032*	
H15B	0.3565	0.8318	0.0592	0.032*	
C16	0.28572 (19)	0.73863 (14)	0.18812 (10)	0.0178 (3)	
H16A	0.1736	0.8094	0.2063	0.021*	
H16B	0.3669	0.7616	0.2212	0.021*	
C17	0.26717 (16)	0.59943 (12)	0.22211 (9)	0.0111 (2)	
H17	0.3818	0.5290	0.2067	0.013*	
C18	0.33000 (18)	0.56602 (13)	0.38753 (10)	0.0174 (3)	
H18A	0.2716	0.5898	0.4558	0.021*	
H18B	0.4065	0.6238	0.3681	0.021*	
C19	0.44121 (17)	0.41627 (14)	0.38303 (10)	0.0158 (3)	
H19A	0.5250	0.4061	0.4250	0.019*	
H19B	0.5064	0.3938	0.3157	0.019*	
C20	0.34221 (17)	0.31229 (13)	0.41426 (9)	0.0138 (3)	
H20	0.2452	0.3333	0.3795	0.017*	
C21	0.2677 (2)	0.32446 (16)	0.52319 (11)	0.0285 (4)	
H21A	0.1971	0.2630	0.5408	0.043*	
H21B	0.1973	0.4188	0.5399	0.043*	
H21C	0.3611	0.2993	0.5587	0.043*	
O1W	0.26626 (14)	−0.02391 (11)	0.47877 (8)	0.0248 (3)	
H1O1	0.3177 (19)	0.0321 (15)	0.4526 (11)	0.030*	
H2O1	0.1784 (15)	0.0183 (16)	0.5179 (10)	0.030*	
O2W	0.04284 (15)	0.88477 (11)	0.39664 (9)	0.0291 (3)	
H1O2	0.075 (2)	0.8012 (10)	0.3828 (13)	0.035*	
H2O2	0.1220 (18)	0.9029 (17)	0.4144 (13)	0.035*	

### Atomic displacement parameters (Å^2^)

**Table:**

	*U*^11^	*U*^22^	*U*^33^	*U*^12^	*U*^13^	*U*^23^
N1	0.0154 (6)	0.0140 (5)	0.0119 (6)	−0.0022 (4)	−0.0064 (4)	0.0016 (4)
N2	0.0090 (5)	0.0082 (5)	0.0147 (6)	−0.0034 (4)	−0.0053 (4)	0.0028 (4)
N3	0.0078 (5)	0.0066 (5)	0.0147 (6)	−0.0021 (4)	−0.0045 (4)	0.0016 (4)
N4	0.0142 (5)	0.0141 (5)	0.0116 (6)	−0.0064 (4)	−0.0020 (4)	−0.0014 (4)
C1	0.0113 (6)	0.0118 (6)	0.0119 (6)	−0.0028 (5)	−0.0032 (5)	0.0016 (4)
C2	0.0135 (6)	0.0198 (7)	0.0239 (8)	0.0017 (5)	−0.0082 (5)	0.0023 (5)
C3	0.0152 (7)	0.0209 (7)	0.0294 (9)	0.0037 (6)	−0.0026 (6)	−0.0031 (6)
C4	0.0217 (7)	0.0134 (7)	0.0253 (8)	0.0028 (6)	−0.0019 (6)	−0.0053 (5)
C5	0.0195 (7)	0.0122 (6)	0.0161 (7)	−0.0014 (5)	−0.0041 (5)	−0.0025 (5)
C6	0.0122 (6)	0.0068 (5)	0.0131 (6)	−0.0016 (5)	−0.0035 (5)	0.0014 (4)
C7	0.0095 (6)	0.0082 (5)	0.0111 (6)	−0.0016 (4)	−0.0032 (4)	0.0013 (4)
C8	0.0145 (6)	0.0126 (6)	0.0253 (8)	−0.0085 (5)	−0.0082 (5)	0.0037 (5)
C9	0.0119 (6)	0.0171 (7)	0.0273 (8)	−0.0080 (5)	−0.0073 (5)	0.0045 (5)
C10	0.0105 (6)	0.0126 (6)	0.0184 (7)	−0.0034 (5)	−0.0075 (5)	0.0010 (5)
C11	0.0119 (7)	0.0200 (7)	0.0338 (9)	−0.0015 (5)	−0.0084 (6)	0.0027 (6)
C12	0.0118 (6)	0.0065 (6)	0.0133 (6)	−0.0019 (5)	−0.0038 (5)	0.0009 (4)
C13	0.0286 (8)	0.0143 (6)	0.0123 (7)	−0.0080 (6)	−0.0071 (5)	0.0028 (5)
C14	0.0424 (9)	0.0170 (7)	0.0160 (7)	−0.0114 (7)	−0.0022 (6)	0.0048 (5)
C15	0.0417 (9)	0.0217 (8)	0.0187 (8)	−0.0203 (7)	0.0049 (7)	−0.0003 (6)
C16	0.0246 (7)	0.0137 (6)	0.0169 (7)	−0.0113 (6)	0.0006 (5)	−0.0017 (5)
C17	0.0117 (6)	0.0099 (6)	0.0113 (6)	−0.0037 (5)	−0.0004 (5)	−0.0012 (4)
C18	0.0241 (7)	0.0160 (7)	0.0154 (7)	−0.0077 (6)	−0.0080 (5)	−0.0029 (5)
C19	0.0167 (7)	0.0173 (7)	0.0167 (7)	−0.0072 (5)	−0.0077 (5)	0.0002 (5)
C20	0.0146 (6)	0.0150 (6)	0.0123 (7)	−0.0048 (5)	−0.0032 (5)	−0.0008 (5)
C21	0.0408 (10)	0.0258 (8)	0.0164 (8)	−0.0129 (7)	0.0047 (7)	−0.0028 (6)
O1W	0.0230 (6)	0.0241 (6)	0.0268 (6)	−0.0091 (5)	−0.0009 (5)	0.0011 (4)
O2W	0.0286 (6)	0.0219 (6)	0.0355 (7)	−0.0030 (5)	−0.0075 (5)	−0.0120 (5)

### Geometric parameters (Å, º)

**Table:**

N1—C1	1.4744 (17)	C10—C11	1.5253 (19)
N1—C20	1.4786 (17)	C10—H10	1.0000
N1—H1N1	0.914 (15)	C11—H11A	0.9800
N2—C7	1.4526 (16)	C11—H11B	0.9800
N2—C8	1.4618 (16)	C11—H11C	0.9800
N2—C6	1.4731 (16)	C12—C13	1.5327 (19)
N3—C7	1.4621 (16)	C12—C17	1.5414 (17)
N3—C10	1.4745 (16)	C12—H12	1.0000
N3—C12	1.4754 (16)	C13—C14	1.5278 (19)
N4—C18	1.4718 (17)	C13—H13A	0.9900
N4—C17	1.4747 (17)	C13—H13B	0.9900
N4—H1N4	0.882 (16)	C14—C15	1.523 (2)
C1—C2	1.5307 (18)	C14—H14A	0.9900
C1—C6	1.5403 (17)	C14—H14B	0.9900
C1—H1	1.0000	C15—C16	1.523 (2)
C2—C3	1.517 (2)	C15—H15A	0.9900
C2—H2A	0.9900	C15—H15B	0.9900
C2—H2B	0.9900	C16—C17	1.5287 (18)
C3—C4	1.521 (2)	C16—H16A	0.9900
C3—H3A	0.9900	C16—H16B	0.9900
C3—H3B	0.9900	C17—H17	1.0000
C4—C5	1.5258 (19)	C18—C19	1.5344 (19)
C4—H4C	0.9900	C18—H18A	0.9900
C4—H4D	0.9900	C18—H18B	0.9900
C5—C6	1.5375 (19)	C19—C20	1.5375 (19)
C5—H5A	0.9900	C19—H19A	0.9900
C5—H5B	0.9900	C19—H19B	0.9900
C6—H6	1.0000	C20—C21	1.5285 (19)
C7—H7A	0.9900	C20—H20	1.0000
C7—H7B	0.9900	C21—H21A	0.9800
C8—C9	1.5238 (19)	C21—H21B	0.9800
C8—H8A	0.9900	C21—H21C	0.9800
C8—H8B	0.9900	O1W—H1O1	0.847 (9)
C9—C10	1.5290 (18)	O1W—H2O1	0.833 (9)
C9—H9A	0.9900	O2W—H1O2	0.837 (9)
C9—H9B	0.9900	O2W—H2O2	0.830 (9)
			
C1—N1—C20	118.35 (10)	C11—C10—H10	108.6
C1—N1—H1N1	106.9 (10)	C9—C10—H10	108.6
C20—N1—H1N1	109.3 (10)	C10—C11—H11A	109.5
C7—N2—C8	109.60 (10)	C10—C11—H11B	109.5
C7—N2—C6	113.54 (10)	H11A—C11—H11B	109.5
C8—N2—C6	114.55 (10)	C10—C11—H11C	109.5
C7—N3—C10	108.08 (9)	H11A—C11—H11C	109.5
C7—N3—C12	112.33 (10)	H11B—C11—H11C	109.5
C10—N3—C12	116.57 (10)	N3—C12—C13	115.37 (10)
C18—N4—C17	115.03 (11)	N3—C12—C17	110.39 (10)
C18—N4—H1N4	111.0 (9)	C13—C12—C17	110.37 (11)
C17—N4—H1N4	105.5 (10)	N3—C12—H12	106.7
N1—C1—C2	108.50 (11)	C13—C12—H12	106.7
N1—C1—C6	112.59 (10)	C17—C12—H12	106.7
C2—C1—C6	109.49 (11)	C14—C13—C12	111.80 (11)
N1—C1—H1	108.7	C14—C13—H13A	109.3
C2—C1—H1	108.7	C12—C13—H13A	109.3
C6—C1—H1	108.7	C14—C13—H13B	109.3
C3—C2—C1	113.15 (12)	C12—C13—H13B	109.3
C3—C2—H2A	108.9	H13A—C13—H13B	107.9
C1—C2—H2A	108.9	C15—C14—C13	110.44 (12)
C3—C2—H2B	108.9	C15—C14—H14A	109.6
C1—C2—H2B	108.9	C13—C14—H14A	109.6
H2A—C2—H2B	107.8	C15—C14—H14B	109.6
C2—C3—C4	111.16 (12)	C13—C14—H14B	109.6
C2—C3—H3A	109.4	H14A—C14—H14B	108.1
C4—C3—H3A	109.4	C16—C15—C14	111.33 (12)
C2—C3—H3B	109.4	C16—C15—H15A	109.4
C4—C3—H3B	109.4	C14—C15—H15A	109.4
H3A—C3—H3B	108.0	C16—C15—H15B	109.4
C3—C4—C5	109.76 (12)	C14—C15—H15B	109.4
C3—C4—H4C	109.7	H15A—C15—H15B	108.0
C5—C4—H4C	109.7	C15—C16—C17	111.59 (11)
C3—C4—H4D	109.7	C15—C16—H16A	109.3
C5—C4—H4D	109.7	C17—C16—H16A	109.3
H4C—C4—H4D	108.2	C15—C16—H16B	109.3
C4—C5—C6	111.95 (12)	C17—C16—H16B	109.3
C4—C5—H5A	109.2	H16A—C16—H16B	108.0
C6—C5—H5A	109.2	N4—C17—C16	110.67 (10)
C4—C5—H5B	109.2	N4—C17—C12	109.40 (10)
C6—C5—H5B	109.2	C16—C17—C12	109.47 (11)
H5A—C5—H5B	107.9	N4—C17—H17	109.1
N2—C6—C5	113.91 (11)	C16—C17—H17	109.1
N2—C6—C1	111.74 (10)	C12—C17—H17	109.1
C5—C6—C1	109.61 (10)	N4—C18—C19	116.65 (11)
N2—C6—H6	107.1	N4—C18—H18A	108.1
C5—C6—H6	107.1	C19—C18—H18A	108.1
C1—C6—H6	107.1	N4—C18—H18B	108.1
N2—C7—N3	109.89 (10)	C19—C18—H18B	108.1
N2—C7—H7A	109.7	H18A—C18—H18B	107.3
N3—C7—H7A	109.7	C18—C19—C20	114.94 (11)
N2—C7—H7B	109.7	C18—C19—H19A	108.5
N3—C7—H7B	109.7	C20—C19—H19A	108.5
H7A—C7—H7B	108.2	C18—C19—H19B	108.5
N2—C8—C9	108.58 (10)	C20—C19—H19B	108.5
N2—C8—H8A	110.0	H19A—C19—H19B	107.5
C9—C8—H8A	110.0	N1—C20—C21	107.37 (11)
N2—C8—H8B	110.0	N1—C20—C19	114.21 (11)
C9—C8—H8B	110.0	C21—C20—C19	110.67 (12)
H8A—C8—H8B	108.4	N1—C20—H20	108.1
C8—C9—C10	112.39 (11)	C21—C20—H20	108.1
C8—C9—H9A	109.1	C19—C20—H20	108.1
C10—C9—H9A	109.1	C20—C21—H21A	109.5
C8—C9—H9B	109.1	C20—C21—H21B	109.5
C10—C9—H9B	109.1	H21A—C21—H21B	109.5
H9A—C9—H9B	107.9	C20—C21—H21C	109.5
N3—C10—C11	112.80 (11)	H21A—C21—H21C	109.5
N3—C10—C9	107.26 (10)	H21B—C21—H21C	109.5
C11—C10—C9	110.94 (12)	H1O1—O1W—H2O1	108.9 (15)
N3—C10—H10	108.6	H1O2—O2W—H2O2	107.8 (15)
			
C20—N1—C1—C2	153.36 (11)	C12—N3—C10—C9	−171.80 (10)
C20—N1—C1—C6	−85.30 (13)	C8—C9—C10—N3	−55.43 (14)
N1—C1—C2—C3	178.89 (12)	C8—C9—C10—C11	−179.03 (12)
C6—C1—C2—C3	55.66 (15)	C7—N3—C12—C13	54.74 (14)
C1—C2—C3—C4	−55.65 (16)	C10—N3—C12—C13	−70.74 (14)
C2—C3—C4—C5	55.09 (16)	C7—N3—C12—C17	−71.21 (12)
C3—C4—C5—C6	−57.78 (16)	C10—N3—C12—C17	163.31 (10)
C7—N2—C6—C5	−51.09 (14)	N3—C12—C13—C14	177.16 (11)
C8—N2—C6—C5	75.88 (14)	C17—C12—C13—C14	−56.88 (15)
C7—N2—C6—C1	73.80 (13)	C12—C13—C14—C15	55.58 (17)
C8—N2—C6—C1	−159.24 (11)	C13—C14—C15—C16	−55.15 (17)
C4—C5—C6—N2	−175.37 (11)	C14—C15—C16—C17	57.13 (17)
C4—C5—C6—C1	58.62 (14)	C18—N4—C17—C16	−89.72 (13)
N1—C1—C6—N2	56.17 (14)	C18—N4—C17—C12	149.58 (10)
C2—C1—C6—N2	176.96 (10)	C15—C16—C17—N4	−178.08 (11)
N1—C1—C6—C5	−176.58 (10)	C15—C16—C17—C12	−57.42 (15)
C2—C1—C6—C5	−55.80 (13)	N3—C12—C17—N4	−53.01 (13)
C8—N2—C7—N3	66.00 (13)	C13—C12—C17—N4	178.27 (10)
C6—N2—C7—N3	−164.48 (10)	N3—C12—C17—C16	−174.44 (10)
C10—N3—C7—N2	−67.79 (12)	C13—C12—C17—C16	56.85 (13)
C12—N3—C7—N2	162.22 (10)	C17—N4—C18—C19	−69.66 (15)
C7—N2—C8—C9	−57.21 (14)	N4—C18—C19—C20	−59.72 (16)
C6—N2—C8—C9	173.83 (11)	C1—N1—C20—C21	176.50 (12)
N2—C8—C9—C10	53.63 (15)	C1—N1—C20—C19	−60.40 (15)
C7—N3—C10—C11	−176.94 (11)	C18—C19—C20—N1	169.30 (11)
C12—N3—C10—C11	−49.35 (15)	C18—C19—C20—C21	−69.41 (15)
C7—N3—C10—C9	60.60 (13)		

### Hydrogen-bond geometry (Å, º)

**Table:**

*D*—H···*A*	*D*—H	H···*A*	*D*···*A*	*D*—H···*A*
N1—H1*N*1···O1*W*^i^	0.914 (15)	2.395 (15)	3.2763 (19)	162.0 (13)
N4—H1*N*4···N3	0.882 (16)	2.287 (15)	2.8304 (16)	119.8 (12)
O1*W*—H1*O*1···N1	0.85 (1)	2.06 (1)	2.9077 (18)	178 (2)
O1*W*—H2*O*1···O2*W*^ii^	0.83 (1)	1.99 (1)	2.816 (2)	175 (2)
O2*W*—H1*O*2···N4	0.84 (1)	2.10 (1)	2.9190 (18)	168 (2)
O2*W*—H2*O*2···O1*W*^iii^	0.83 (1)	1.98 (1)	2.7975 (17)	166 (2)

Symmetry codes: (i) −*x*+1, −*y*, −*z*+1; (ii) −*x*, −*y*+1, −*z*+1; (iii) *x*, *y*+1, *z*.
